# Network oscillatory dynamics accompany cerebral bioenergetic defence in hypoxia

**DOI:** 10.1177/0271678X261447119

**Published:** 2026-06-04

**Authors:** Damian M Bailey, Benjamin S Stacey, Yaopeng Ma, Takuro Washio, Hayato Tsukamoto, Thomas S Owens, Thomas A Calverley, Lewis Fall, Christopher J Marley, Angelo Iannetelli, Takeshi Hashimoto, Soichi Ando, Shigehiko Ogoh, Nicola Marchi, Josip Butkovic, Ivan Mumlek, Brad Parry, Zvonomir Vrselja, James A Pawelczyk, Ronny P Bartsch

**Affiliations:** 1 Neurovascular Research Laboratory, Faculty of Life Sciences and Education, University of South Wales, Pontypridd, UK; 2 Bexorg, Inc., New Haven, CT, USA; 3 Keck Laboratory for Network Physiology, Department of Physics, Boston University, Boston, MA, USA; 4 Institute for Exercise and Environmental Medicine, Texas Health Presbyterian Hospital Dallas, The University of Texas Southwestern Medical Center, Dallas, TX, USA; 5 Faculty of Sport Sciences, Waseda University, Tokorozawa, Japan; 6 Faculty of Sport and Health Science, Ritsumeikan University, Shiga, Japan; 7 Graduate School of Informatics and Engineering, The University of Electro-Communications, Tokyo, Japan; 8 Department of Biomedical Engineering, Toyo University, Asaka, Japan; 9 CNRS 5203, INSERM U1191, Institute of Functional Genomics, University of Montpellier, Montpellier, France; 10 Noll Laboratory, Department of Kinesiology, Pennsylvania State University, University Park, PA, USA; 11 Department of Physics, Bar-Ilan-University, Ramat Gan, Israel

**Keywords:** Hypoxia, acute mountain sickness, network physiology, cerebral substrate delivery, very low-frequency oscillations

## Abstract

A network physiology framework investigated how coordinated interactions among multiple organ systems collectively support the preservation of cerebral bioenergetic function and better distinguish adaptive from maladaptive responses to hypoxia. Twelve healthy males were passively exposed to 6 h of normoxia (21% O_2_) and hypoxia (12% O_2_) in a randomised, single-blind, crossover design. Venous blood was assayed for oxidative-nitrosative stress (OXNOS, spectroscopy/chemiluminescence) and neurovascular unit (hs-ELISA) biomarkers. Global cerebral delivery of O_2_ and glucose were determined by duplex ultrasound. Clinical acute mountain sickness (AMS+) was diagnosed in five participants. Cerebral substrate delivery was well maintained in both hypoxia and AMS+ (*p* < 0.05 vs normoxia and AMS−) despite marked arterial hypoxemia. Bioenergetic defence coincided with pronounced elevations in the spectral amplitude and phase synchronisation of very low-frequency oscillations (VLFOs, 0.03–0.06 Hz), which were evident across multiple organ systems and most prominent within the cerebral network. Systemic VLFOs were further exaggerated and more functionally connected in AMS+ in the absence of exaggerated systemic OXNOS or structural damage/destabilisation of the neurovascular unit (both *p* < 0.05 vs normoxia and AMS−). Collectively, these findings suggest that AMS, while characterised by debilitating symptomatology, may reflect a neuroprotective adaptive as opposed to pathologically maladaptive phenotype.

## Introduction

The ability to sense and initiate corrective adjustments to maintain cellular oxygen (O_2_) and carbon dioxide (CO_2_) homeostasis is essential for the survival of all respiring organisms, to which the human brain has evolved exquisite sensitivity.^1–3^ Cerebral vasodilatation to hypoxia and hypercapnia with reciprocal vasoconstriction to hyperoxia and hypocapnia are fundamental, highly conserved physiological responses that serve to couple cerebral O_2_ delivery (CDO_2_) and CO_2_ removal to tissue metabolic demand.^4–6^ This is crucial for survival given the brain’s inherent vulnerability to hypoxia, predicated on its obligatory high rate of O_2_ consumption in the face of limited glycolytic reserves,^7^ with heightened reactivity to altered CO_2_ reflecting prioritisation of acid-base balance for stabilisation of chemosensory and autonomic control at the level of the brainstem.^4,8^ However, despite intense research efforts, the integrative multi-organ mechanisms that sense crosstalk between these respiratory gases, including the coordinated transmission of adaptive signals that collectively preserve cerebral bioenergetic homeostasis, remain elusive.^9^

Failure to make adequate adjustments to hypoxia can further compound hypoxemia and increase vulnerability to the maladaptive neurological syndrome of acute mountain sickness (AMS) at terrestrial high-altitude (HA).^10^ Characterised by headache and associated vegetative symptoms, AMS is typically experienced by non-acclimatised mountaineers within 6–12 h of ascent above 2500 m.^11^ While benign, AMS may progress in severe cases and with continued ascent, to high-altitude cerebral oedema, that if left untreated, can result in death due to brain herniation.^10^ While the underlying pathophysiology remains controversial, functional impairments in pulmonary, cerebrovascular, autonomic and nociceptive reactivity – combined with free radical–mediated reductions in vascular nitric oxide (NO) bioavailability, collectively termed oxidative–nitrosative stress (OXNOS) – may compromise the integrity of the neurovascular unit, predispose to intracranial hypertension, and have been recognised as potential risk factors.^10,12–15^

The emerging interdisciplinary field of network physiology (NP) has the potential to provide complementary insight into system-wide integrative mechanisms to better differentiate physiological adaptation from pathological maladaptation to hypoxia. Originally developed by physicists, NP moves beyond traditional univariate, organ-centric analyses by explicitly examining how multiple organ systems – each with their own complex structure and regulatory mechanisms – synchronise and coordinate their output dynamics as a global, integrated network, thereby providing insight into emergent physiological behaviour and phenotypic plasticity.^16,17^

Herein, we applied a NP framework to understand how acute hypoxia impacts the temporal dynamics and functional interactions between the cerebral, cardiac, pulmonary and metabolic systems. Having identified a priori that hypoxia was associated with enhanced spectral power density of very low-frequency oscillations (VLFOs, 0.03–0.06 Hz) across multi-organ systems, we sought to determine if hypoxia (primary aim) and corresponding susceptibility to AMS (secondary aim) were associated with distinct network phenotypes, characterised by discrete topologies, node connectivities, numbers and strengths of links. We hypothesised that VLFOs would be^1^ amplified and more synchronised in hypoxia compared to normoxia, collectively reflecting greater connection of the integrative responses that preserve global cerebral substrate delivery of O_2_ and glucose (CDO_2_/CD_Glu_) in the face of elevated systemic OXNOS, and^2^ more suppressed and desynchronised in participants who develop AMS (AMS+) compared to those without (AMS−) and linked to impaired CDO_2_/CD_Glu_ and structural destabilisation of the neurovascular unit subsequent to exaggerated systemic OXNOS, reflecting the dynamic transition from a physiologically adaptive to pathologically maladaptive neurological phenotype.

## Materials and methods

### Ethical approval

The experimental protocol was approved by the Research Ethics Committees of the University of South Wales (#201712BS01). All experimental procedures were carried out in accordance with the Declaration of Helsinki of the World Medical Association^18^ with the exception of registration in a database. Verbal and written informed consent were obtained from all participants.

### Participants

We recruited 12 healthy, physically active males aged 23 (mean) ± 2 (SD) years with a body mass index of 25 ± 4 kg/m^2^ and body fat of 15% ± 7% who resided permanently at ~183 m above sea level in the local region surrounding the University of South Wales, UK. Participants did not receive any financial compensation for their involvement in the study. All participants were non-smokers, not prescribed any medications and abstained from taking nutritional supplements, including oral antioxidants and anti-inflammatories. Participants were specifically asked to refrain from physical activity, caffeine, alcohol and high-fat meals for a period of 48 h prior to formal experimentation, consistent with our previous approaches designed to minimise biological variation.^19,20^ They were also encouraged to follow a low-nitrate/nitrite diet for 96 h prior to the study, with specific instructions to avoid fruits, salads and cured meats.^21^

### Design

Select cardiopulmonary and cerebrovascular metrics have been published as part of separate investigations focussed on the impact of hypoxia on the respiratory chemoreflex^22^ and cognition.^23^ Thus, although the present study adopted an identical experimental design (randomised single-blinded, counterbalanced two-period, two-treatment cross-over trial), it constitutes an entirely different investigation focussed on distinctly different primary end-outcome variables (network connections) and complementary de novo metrics of systemic OXNOS and neurovascular unit biomarkers. Participants completed two separate experimental trials in a normobaric environmental chamber (~120 m^3^) maintained at 21 °C and 50% relative humidity (Design Environmental, Ebbw Vale, UK) using a computer-generated block randomisation procedure to ensure equal sequence allocation. Six participants completed the AB sequence (normoxia → hypoxia) and six completed the BA sequence (hypoxia → normoxia) for the first exposure.

Each trial involved 6 h passive exposure to normoxia (21% O_2_) and normobaric hypoxia (12% O_2_), achieved by altering the inspired fraction of O_2_ (F_I_O_2_) under the prevailing barometric pressure (759 ± 5 mmHg, range: 732–679 mmHg for the duration of the study period). Hypoxic air was generated using a molecular sieve–based gas separation system, which selectively removes nitrogen from compressed ambient air via pressure swing adsorption, thereby enriching the remaining gas stream with nitrogen and reducing the F_I_O_2_ to the desired level. This normobaric hypoxic stimulus corresponds to an equivalent terrestrial altitude of ~4500 m, a level at which unacclimatised individuals are at appreciable risk of developing AMS. Exposure was acute (i.e. without prior acclimation) and typically results in arterial PO_2_ values of ~40 mmHg, comparable to those observed in patients with advanced cardiopulmonary disease.

Each trial was separated by a ⩾7-day washout period, considered a priori sufficient to minimise physiologically meaningful hypoxia-related carryover beyond normal basal variation in select measures of systemic OXNOS and cardiopulmonary–cerebrovascular function.^24,25^ With the exception of AMS and headache scores that were recorded hourly, all NP, molecular and haemodynamic metrics were documented after 6 h exposures to coincide with clinical diagnosis of AMS. Participants were instructed to arrive at the laboratory following a 12 h overnight fast and consumed a standardised meal (30 g of oats with 180 mL water) at the following time points: 30 min prior to testing and following 2, 4 and 6 h in normoxia/hypoxia to maintain hunger comfort and hydration. All participants, including those who developed AMS+, consumed the full standardised meal at each time point; no incomplete intake was recorded.

### Blood sampling

Blood was obtained without stasis from an indwelling cannula located in a forearm antecubital vein into Vacutainers^®^ (Becton, Dickinson and Company, Oxford, UK) before immediate centrifugation at 600*g* (4 °C) for 10 min. Serum, plasma and red blood cell (RBC) samples were decanted into cryogenic vials (Nalgene^®^ Labware; Thermo Fisher Scientific, Inc., Waltham, MA, USA) and snap-frozen under liquid nitrogen prior to storage at −80 °C. Samples were left to defrost at 37 °C in the dark for 5 min before batch analysis.

### Measurements

#### Molecular function

##### Haematology

Haemoglobin (Hb) was measured photometrically (HemoCue 201+; Radiometer, UK).^26^ Haematocrit (Hct) was assessed via ultracentrifugation (Hawksley and Sons Ltd., Sussex, UK) and measured using a Hawksley Micro Hematocrit Reader (Hawksley and Sons Ltd., Sussex, UK). For both Hb and Hct, triplicate samples were obtained and the mean value used for overall analysis. Hb and peripheral oxygen saturation (SpO_2_) were used to estimate arterial oxygen content (caO_2_, see “Cerebral bioenergetics” section). Glucose was assessed photometrically in triplicate and the average calculated (Randox Daytona Plus; Randox, Country Antrim, UK). Intra- and inter-assay co-efficients of variation (CVs) for all measured metabolites were both <5%.

##### Systemic OXNOS: Ascorbate free radical (A^·−^)

We employed electron paramagnetic resonance (EPR) spectrosopic detection of A^·−^ as a direct measure of global systemic free radical formation.^27^ Plasma (1 mL) was injected directly into a high-sensitivity multiple-bore sample cell (AquaX; Bruker Daltonics, Inc., Billerica, MA, USA) housed within a TM_110_ cavity of an EPR spectrometer operating at X-band frequency (9.87 GHz). Samples were recorded by cumulative signal averaging of 10 scans using the following instrument parameters: resolution, 1024 points: microwave power, 20 mW; modulation amplitude, 0.65 G; receiver gain, 2 × 10^6^; time constant, 40.96 ms; sweep rate, 0.14 G/s; sweep width, 6 G; centre field, 3486 G. All spectra were filtered identically (moving average, 15 conversion points) using WINEPR software (Version 2.11; Bruker, Karlsruhe, Germany) and the double integral of each doublet quantified using Origin 8 software (OriginLab Corps, MA, USA). Intra- and inter-assay CVs were both <5%.^27^

##### Nitric oxide (NO) metabolites

The modified triiodide ( $I_{3}^{-}$ )-based chemiluminescence assay (Sievers NOA 280i; Analytix Ltd, Durham, UK) was employed to detect (total) plasma and red blood cell (RBC)-bound NO. Plasma (200 μL) was injected into acidified $I_{3}^{-}$ reducing reagent for the combined measurement of nitrite $\left(NO_{2}^{-}\right)$ and *S*-nitrosothiols. RBCs (250 µL) were lysed 1:4 with EDTA (0.5 mM; pH corrected to 7.0) and incubated for 5 min on ice. RBC lysate (400 µL) was injected into modified $I_{3}^{-}$ reagent containing potassium hexacyanoferrate to limit NO auto-capture by deoxygenated Hb/cell-free heme, for the combined measurement of RBC-bound NO reflecting the cumulative concentration of $NO_{2}^{-}$ , *S*-nitrosohaemoglobin (SNO-Hb) and iron nitrosylhaemoglobin (HbNO).^28–30^ Total NO concentration was calculated as the cumulative concentration of plasma and RBC NO metabolites. Signal output was plotted against time using Origin 8 software (OriginLab Corps, MA, USA) and smoothed using a 150-point averaging algorithm. The Peak Analysis package was used to calculate the area under the curve and subsequently converted to a concentration using standard curves of sodium $NO_{2}^{-}$ . Intra- and inter-assay CVs for all measured metabolites were both <10%.^28^

##### Neurovascular unit integrity

We adopted a molecular approach focussing on blood-borne neurovascular unit–specific proteins.^31^ Serum S100B, a calcium-binding protein expressed predominantly by astrocytes and Schwann cells found at the perivascular brain space,^32^ was employed as a surrogate biomarker of BBB permeability. While its appearance in the systemic circulation has been shown to correlate with the extent and temporal sequence of BBB opening, interpretation is constrained by delayed systemic appearance, continuous basal cerebral release, potential extracranial sources and the absence of serial or arterio-jugular venous sampling (precluded by ethical and logistical constraints).^33^ Neuron-specific enolase (NSE), an intracytoplasmic glycolytic enzyme derived from neuronal cytoplasm and neuroendocrine cells,^34^ was employed as a biomarker of neuronal injury and is similarly subject to delayed systemic kinetics. Both proteins were quantified using automated high-sensitivity clinical-grade ELISA (LIAISON^®^; DiaSorin, Saluggia, Italy). Intra- and inter-assay CVs for all metabolites were <5%.

#### Cardiopulmonary function

A three-lead electrocardiogram ((ECG) ADI BioAmp ML132) was used to assess heart rate (HR). Finger photoplethysmography (Finometer PRO; Finapres Medical Systems, Amsterdam, The Netherlands) was used to measure beat-by-beat blood pressure, stroke volume (SV) and cardiac output ( $\overset{\cdot }{Q}$ ) using the Modelflow algorithm^35^ that incorporates participant sex, age, stature and mass (BeatScope 1.0 software; TNO; TPD Biomedical Instrumentation, Amsterdam, The Netherlands). The blood pressure waveform was used to calculate mean arterial pressure (MAP) after calibrating values to the average of two automated brachial blood pressure measurements (Life Source; A&D Medical, model: UA767FAM) taken over a 5-minute resting baseline period. Total peripheral resistance (TPR) was calculated as MAP/ $\overset{\cdot }{Q}$ . Respiratory gases (O_2_ and CO_2_) were sampled continuously at the mouth via capnography (model ML206; AD Instruments) calibrated daily to atmospheric pressure for determination of end-tidal partial pressures of O_2_ and CO_2_ (P_ET_O_2_/P_ET_CO_2_). Minute ventilation ( ${\overset{\cdot }{V}}_{E}$ ) was determined using a pneumotachometer (model HR 800 L; Hans Rudolph, Shawnee, KS, USA) and SpO_2_ was recorded continuously by pulse oximetry (Nonin 9550 Onyx II; Nonin Medical, Inc., Plymouth, MI, USA).

#### Cerebrovascular function

##### Intracranial perfusion

Continuous assessments of blood velocity in the middle cerebral artery (MCAv), insonated through the left temporal window and posterior cerebral artery (PCAv), insonated at the P1 segment through the right temporal window, were measured using standardised procedures with a 2 MHz pulsed transcranial Doppler ultrasound probe (Multi-Dop X4; DWL Elektronische Systeme GmbH, Sipplingen, Germany).

##### Extracranial perfusion

Contralateral continuous assessments of diameter, velocity and blood flow recordings in the right internal carotid and left vertebral arteries ${\overset{\cdot }{Q}}_{\mathrm{ICA}}$ and ${\overset{\cdot }{Q}}_{\mathrm{VA}}$ were obtained using a 10 MHz, multifrequency, linear array vascular ultrasound (Terason 3200; Teratech, Burlington, MA, USA). Arterial diameter was measured via B-mode imaging, whereas peak blood velocity was simultaneously measured with pulse-wave mode. The ICA was insonated ⩾1.5 cm from the carotid bifurcation, with no evidence of turbulent or retrograde flow present during recording. The VA was insonated at the C4–C5 or C5–C6 vertebral segment and standardised within participants for both trials. The steering angle was fixed to 60° and the sample volume was placed in the centre of the vessel and adjusted to cover the entire vascular lumen. All images were recorded as video files at 30 Hz and stored for offline analysis using customised edge detection software designed to mitigate observer bias.^36^ Simultaneous measures of arterial diameter and velocity over >12 consecutive cardiac cycles were used to calculate flow. Between-day CVs for ${\overset{\cdot }{Q}}_{\mathrm{ICA}}$ and ${\overset{\cdot }{Q}}_{\mathrm{VA}}$ are 5% and 11%, respectively.^37^

##### Cerebral bioenergetics

Volumetric blood ( ${\overset{\cdot }{Q}}_{\mathrm{ICA}}$ _ICA_ and ${\overset{\cdot }{Q}}_{\mathrm{VA}}$ _VA_, mL/min) was calculated offline as:

$$\frac{ICA\;Or\;VAV_{P}(cm/s)}{2}\times \pi {\left(\frac{ICA\;Or\;VA\;Diameter(cm)}{2}\right)}^{2}\times 60$$

where V_p_ is peak envelope blood velocity.

Acknowledging unilateral measurement errors when assuming symmetrical blood flow of contralateral ICA and VA arteries,^38^ global cerebral blood flow (gCBF) was calculated as:

$$gCBF(mL/\min)=2\times ({\overset{\cdot }{Q}}_{ICA}+{\overset{\cdot }{Q}}_{VA})$$

Cerebrovascular conductance (CVCi) indices were calculated as:

$$\begin{array}{l} CVCi(mL/\min/mmHg)=\\ \frac{\begin{array}{l} MCAv\;Or\;PCAv\left(cm/s\right)Or\;{\overset{\cdot }{Q}}_{ICA}Or\\ {\overset{\cdot }{Q}}_{VA}Or\;gCBF(mL/miN) \end{array}}{MAP(mmHg)} \end{array}$$

Estimated arterial oxygen content (caO_2_, mL/dL) was estimated as:

$$\begin{array}{l} \left(Hb(g/dL)\times 1.34\times \frac{SpO_{2(\%)}}{100}\right)+\\ {}[0.003\times PaO_{2}(mmHg)]. \end{array}$$

where 1.34 is the O_2_ binding capacity of Hb, 0.003 is the solubility of O_2_ dissolved in blood and assuming an SpO_2_ of 97% and arterial PO_2_ (PaO_2_) of 100 mmHg.

Cerebral substrate delivery of oxygen and glucose (DO_2_ and D_Glu_) were calculated as:

$$\begin{array}{l} MCAv/PCAv-DO_{2}(mL/\min)=\\ MCAv/PCAv(cm/s)\times caO_{2}(mL/dL) \end{array}$$

$$\begin{array}{l} MCAv/PCAv-D_{Glu}(mL/\min)=\\ MCAv/PCAv(cm/s)\times glucOse(mmOl/L) \end{array}$$

$$\begin{array}{l} ICA/VA-DO_{2}(mL/\min)=\\ \left(\frac{\overset{\cdot }{Q}ICA/{\overset{\cdot }{Q}}_{VA}(mL/miN)}{100}\right)\times caO_{2}(mL/dL) \end{array}$$

$$\begin{array}{l} ICA/VA-D_{Glu}(mL/\min)=\\ \left(\frac{\overset{\cdot }{Q}ICA/{\overset{\cdot }{Q}}_{VA}(mL/miN}{1000}\right)\times glucOse(mmOl/L) \end{array}$$

#### Clinical function

##### Acute mountain sickness (AMS) and headache

Neurological symptoms ascribed to AMS were examined hourly using the newly revised Lake Louise (LL)^39^ and Environmental Symptoms Questionnaires-Cerebral Symptoms (ESQ-C)^40^ questionnaires. Participants were also asked to rate their cephalalgia using a clinically validated visual analogue scale (0–100 mm; 0 mm = no headache, 10 mm = mild headache including a sensation of pressing or throbbing, 50 mm = moderate intensity headache and 100 mm = worst possible headache).^41^ Clinical (moderate-to-severe) AMS was diagnosed if a participant presented with a combined total LL score of ⩾ 5 points in the presence of a headache and ESQ-C score ⩾0.700 points at the 6 h exposure to hypoxia timepoint.^42^ Based on previous observations, we anticipated that this would result in ~50% of the participants (*n* = 6) developing moderate-to-severe AMS (AMS+) with the remainder staying healthy (AMS−).^42^

#### Data integration

With the exception of extracranial blood flow measures (discrete sampling), all cardiopulmonary and (intracranial) cerebrovascular variables were sampled continuously at 1 kHz using an analogue-to-digital converter (Powerlab, 16/30; AD Instruments, Colorado Springs, CO, USA) without filtering and data were interfaced with LabChart (Version 7.1) and analysed offline. This procedure ensured that all signals follow a common clock, which is important for synchronisation analysis and obtaining physiological networks.

#### Network physiology (NP)

NP interrogates the coupling and dynamic interactions among organ systems by analysing synchronous recordings of key physiologic parameters and output signals from multiple systems, thus providing a more integrated approach compared to more traditional, reductionist, single-signal organ-centric analysis.^16,17,43^ Herein, we identified that acute hypoxia was associated with VLFOs in all (intracranial) cerebrovascular and cardiopulmonary variables. We computed a network inference from the extracted signals and subsequently compared how these were altered in AMS.

##### VLFOs

All (intracranial) cerebrovascular and cardiopulmonary data were subject to Fourier analysis to assess power spectral density. When comparing densities, we observed that the physiological signals demonstrated enhanced power in the VLF band (0.03–0.06 Hz) during hypoxia. To quantify physiological changes during the transition from normoxia to hypoxia, we computed the relative power (RP) for each signal using:

$$\mathrm{RP}=\ln\left(\frac{\mathrm{VLF}\;\mathrm{power}\;\mathrm{in}\;\mathrm{hypoxia}}{\mathrm{VLF}\;\mathrm{power}\;\mathrm{in}\;\mathrm{normoxia}}\right)$$

Thus, a relative power equal to 0 reflects no change, and a relative power >0 reflects increased VLFO amplitude in hypoxia. To further investigate the hypoxic influence on physiological interactions, we extracted the VLFO using a Fourier transform bandpass filter. Specifically, each signal was first transformed into the frequency domain via the fast Fourier transform (FFT). Spectral components within the range of 0.03–0.06 Hz were retained, while all other frequencies were set to zero. The filtered signal was then reconstructed through the inverse Fourier transform, yielding the VLFO component that was subsequently used for further analyses.

##### Network links

Next, to quantify synchronisation between physiological measurements, we applied phase synchronisation analysis. The analytic signal of each VLFO time series x(t) was derived using the analytic signal approach and Hilbert transform:

$$s(t)=x(t)+{\mathrm{ix}}_{H}(t)={A(t)e}^{i\phi (t)}$$

where x_H_(t) is the Hilbert transform of x(t), and φ(t) is the instantaneous phase of the signal.

For any two signals n and m, their instantaneous phase difference is defined as:

$$\psi_{\mathrm{nm}}(t)=\phi_{N}(t)—\phi_{m}(t)$$

To define significant physiological coupling (network links) between two signals, we calculated the phase synchronisation index (γ) using:

$$\gamma_{\mathrm{nm}}^{2}={〈\cos\psi_{\mathrm{nm}}(t)〉}^{2}+{〈\sin\psi_{\mathrm{nm}}(t)〉}^{2}$$

where ψ(t) is the instantaneous phase difference of the signals as calculated by the analytic signal approach using the Hilbert transform.^44^

Given that a narrow bandwidth can lead to spurious detection of phase synchronisation, and to correct the upper estimate of phase synchronisation, we established a baseline for uncoupled signals using a surrogate test.^45^ For every signal pair (n, m) surrogate γ distributions were generated by pairing signals taken from two different participants within the same experimental group (normoxia or hypoxia). All possible cross-participant combinations were considered, thereby yielding a distribution of γ values that reflects the level of synchronisation expected by chance. From this surrogate distribution, the fifth percentile value was selected as the significance threshold. For each participant, if the γ-value obtained from the real signal pair (n, m) exceeded its corresponding surrogate threshold, the link was considered physiologically significant and retained in the adjacency matrix. Conversely, if the γ value was below the threshold, the corresponding entry in the adjacency matrix was set to zero, indicating a lack of significant coupling.

Following the identification of significant links, individual adjacency matrices were obtained for each participant under each experimental condition. To derive representative networks, adjacency matrices were averaged within groups. Specifically, matrices from all participants under the same condition (normoxia or hypoxia) were averaged to yield group-level networks representing different oxygen levels. Similarly, matrices were averaged across participants according to state (AMS+ or AMS−), resulting in representative networks for AMS.

### Statistical analysis

#### Prospective power calculations and sample size estimates

Data were analysed using G*Power 3.1 software. *Condition effect (hypoxia vs normoxia)*: Assuming comparable differences and corresponding effect sizes (dz statistic reflecting standardised mean difference) previously observed for select OXNOS (plasma NO ( ${NO}_{2}^{-}$ + RSNO): dz = 1.10), cardiopulmonary (SpO_2_: dz = 5.33), cerebrovascular (gCBF: dz = 0.87) and AMS (LL: dz = 1.94/ESQ-C: dz = 1.19/VAS: dz = 1.26) metrics^15^ and pilot data for phase synchronisation (mean link strength of entire network) metrics (dz = 1.157), the present study required a (minimum) sample size of 7, 6, 10, 4/6/6 and 10 participants, respectively, to achieve a power (1 − β) of 0.80 at *p* < 0.05 for one-tailed tests. *State effect (AMS+ vs AMS*−*)*: Based on previous observations, we anticipated that 50% of our participants (*n* = 6/12) would develop moderate to severe AMS (AMS+) with the remainder staying symptom free (AMS−).^42^ While (between state) differences in SpO_2_ and CBF have not consistently been observed, nor NP metrics tested in the setting of AMS+, the sample size (*n* = 12, 6 vs 6) was considered adequate to detect differences in plasma NO ( ${NO}_{2}^{-}$ + RSNO): dz = 1.73 and AMS symptomatology (LL: dz = 2.11/ESQ-C: dz = 1.92/VAS: dz = 2.04) requiring a total (AMS+ and AMS− combined) of 10 and 8/10/8 participants,^14^ respectively, to achieve a power (1 − β) of 0.80 at *p* < 0.05 for one-tailed tests. We chose to inflate our final sample size to 12 participants given the potential for loss-to-follow-up and/or technical complications.

#### Inferential statistics

Data were analysed using the Statistics Package for Social Scientists (IBM SPSS Statistics Version 29.0) and the SciPy package.^46^ Shapiro–Wilk *W* tests were performed to assess distribution normality. Temporal kinetics of AMS and headache symptoms were analysed using two-way (condition × state) repeated measures analyses of variance. Within condition differences were analysed using paired sample *t*-tests or Wilcoxon matched-pairs signed-rank tests where appropriate. Between state differences were assessed using independent samples *t*-tests or Mann–Whitney *U* tests where appropriate. One-sided Kolmogorov–Smirnov tests were employed to determine between state differences in the cumulative distribution function. Relationships between select variables were determined using Pearson product moment or Spearman rank correlations. Significance was established at *p* < 0.05 and data are expressed as mean ± standard deviation (SD) for all two-tailed tests.

## Results

### Clinical function

Of the 12 participants exposed to hypoxia, five (~42%) were diagnosed with clinical AMS+ and seven participants (~58%) remained healthy (AMS−). Supplementary Figure 1 illustrates the temporal evolution of AMS and corresponding headache scores, that as anticipated, were markedly elevated in hypoxia (Supplementary Figure 1(A)–(C)) and further compounded in AMS+ (Supplementary Figure 1(D)–(F)).

### Molecular function

No changes were observed in Hb, Hct or glucose in hypoxia or AMS+ (Table 1). Although Hct might be expected to increase during acute hypoxia reflecting a haemoconcentration subsequent to a reduction in plasma volume, examination of within-participant changes revealed only modest inter-individual variability (Δ hypoxia–normoxia: 1% ± 3%), with no consistent directional trend. While plasma A^·−^ and NO did not change, hypoxia was associated with a reduction in RBC NO that was more pronounced in AMS+ (Table 1). Hypoxia decreased S100B and NSE whereas no differences were observed in AMS+ (Table 1).

**Table 1.** Molecular function.

	Inspirate			State		
	Normoxia (*n* = 12)	Hypoxia (*n* = 12)	*p* values	Δ AMS− (*n* = 7)	Δ AMS+ (*n* = 5)	*p* values
**Haematology**						
Hb (g/dL)	14.7 ± 1.2	14.7 ± 0.9	0.965	−0.1 ± 1.4	0.1 ± 0.8	0.804
Hct (%)	45 ± 3	46 ± 2	0.346	1 ± 2	0 ± 3	0.489
Glucose (mmol/L)	6.0 ± 0.9	6.4 ± 0.6	0.181	0.4 ± 1.2	0.4 ± 0.5	0.903
**Free radicals**						
A^·−^ × 10^3^ (AU)	331 ± 122	279 ± 93	0.273	−76 ± 178	−18 ± 134	0.554
**NO metabolites**						
Plasma NO (nM)	103 ± 46	93 ± 50	0.574	−15 ± 73	−1 ± 28	0.666
RBC NO (nM)	150 ± 44	101 ± 42	**0.017**	−24 ± 60	−82 ± 43	**0.047**
Total NO (nM)	252 ± 75	195 ± 83	**0.047**	−40 ± 112	−83 ± 46	0.432
**Brain-specific proteins**						
S100B (μg/L)	0.029 ± 0.014	0.023 ± 0.005	**0.046**	−0.008 ± 0.012	−0.003 ± 0.006	0.379
NSE (μg/L)	8.707 ± 1.892	6.717 ± 1.339	**0.008**	−1.999 ± 2.402	−1.978 ± 1.927	0.987

Hb: haemoglobin; Hct: haematocrit; A^−^: ascorbate free radical; NO: nitric oxide; NSE: neuron-specific enolase.

Values are mean ± SD based on pooled data (normoxia vs hypoxia, *n* = 12) and change (Δ hypoxia minus normoxia) in participants diagnosed with and without clinical acute mountain sickness (AMS+, *n* = 5 vs. AMS−, *n* = 7).

Bold values indicate significance of *p*-values < 0.05.

### Cardiopulmonary function

As anticipated, hypoxia decreased P_ET_O_2_, P_ET_CO_2_ and SpO_2_ and were accompanied by a reduction in caO_2_ and MAP (Table 2). These coincided with an elevation in ${\overset{\cdot }{V}}_{E}$ HR and $\overset{\cdot }{Q}$ with no changes observed in SV and reduction in TPR (Table 2). No differences were observed in AMS+ (Table 2).

**Table 2.** Cardiopulmonary function.

	Inspirate			State		
	Normoxia (*n* = 12)	Hypoxia (*n* = 12)	*p* values	Δ AMS− (*n* = 7)	Δ AMS+ (*n* = 5)	*p* values
P_ET_O_2_ (mmHg)	93 ± 5	47 ± 6	<**0.001**	−47 ± 4	−42 ± 7	0.189
P_ET_CO_2_ (mmHg)	43 ± 6	31 ± 4	<**0.001**	−10 ± 7	−14 ± 5	0.196
SpO_2_ (%)	98 ± 1	86 ± 4	<**0.001**	−13 ± 4	−13 ± 3	0.926
caO_2_ (mg/dL)	19.6 ± 1.7	17.1 ± 1.2	<**0.001**	−2.7 ± 2.2	−2.3 ± 1.0	0.698
${\overset{\cdot }{V}}_{E}$ (L/min)	16 ± 6	21 ± 5	**0.034**	3 ± 6	7 ± 11	0.429
HR (bpm)	57 ± 11	73 ± 12	<**0.001**	16 ± 15	17 ± 8	0.862
SV (mL)	97 ± 3	100 ± 1	0.537	0 ± 1	0 ± 1	0.729
*Q* (L/min)	5.7 ± 1.1	7.3 ± 1.2	<**0.0001**	1.6 ± 1.5	1.7 ± 0.8	0.844
MAP (mmHg)	90 ± 7	78 ± 9	**0.003**	−8 ± 12	−17 ± 12	0.225
TPR (mmHg/L/min)	16.4 ± 3.4	11.0 ± 2.4	<**0.001**	−4.9 ± 3.9	−6.0 ± 2.2	0.548

P_ET_O_2_/P_ET_CO_2_: end-tidal partial pressure of oxygen/carbon dioxide; SpO_2_: peripheral arterial oxyhaemoglobin saturation; caO_2_: arterial oxygen content; ${\overset{\cdot }{V}}_{E}$ : pulmonary ventilation; HR: heart rate; SV: stroke volume; $\overset{\cdot }{Q}$ : cardiac output; MAP: mean arterial pressure; TPR: total peripheral resistance.

Values are mean ± SD based on pooled data (normoxia vs hypoxia, *n* = 12) and change (Δ hypoxia minus normoxia) in participants diagnosed with and without clinical acute mountain sickness (AMS+, *n* = 5 vs. AMS−, *n* = 7).

Bold values indicate significance of *p*-values < 0.05.

### Cerebrovascular function

#### Intracranial bioenergetics

Hypoxia did not alter MCAv or PCAv whereas both MCAv- and PCAv-CVCi increased (Table 3) due to the reduction in MAP (Table 2). Hypoxia selectively reduced MCA-CDO_2_ that was more marked in AMS+ subsequent to a greater reduction in MCAv, whereas no changes were observed in CD_Glu_ (Table 3).

**Table 3.** Cerebrovascular function.

	Inspirate			State		
	Normoxia (*n* = 12)	Hypoxia (*n* = 12)	*p* values	Δ AMS− (*n* = 7)	Δ AMS+ (*n* = 5)	*p* values
**Intracranial – anterior (MCA)**						
Velocity (cm/s)	63 ± 12	60 ± 12	0.217	1 ± 7	−9 ± 5	**0.023**
CVCi (cm/s/mmHg)	0.71 ± 0.16	0.78 ± 0.18	0.053	0.09 ± 0.14	0.05 ± 0.16	0.700
CDO_2_ (mL/cm/s)	1230 ± 225	1031 ± 234	**0.002**	−122 ± 168	−305 ± 100	**0.028**
CD_Glu_ (mmol/cm/s)	380 ± 95	384 ± 75	0.855	29 ± 86	−30 ± 55	0.179
I**ntracranial – posterior (PCA)**						
Velocity (cm/s)	36 ± 4	37 ± 9	0.635	2 ± 5	−1 ± 9	0.521
CVCi (cm/s/mmHg)	0.40 ± 0.05	0.48 ± 0.10	**0.023**	0.07 ± 0.10	0.08 ± 0.10	0.920
CDO_2_ (mL/cm/s)	705 ± 97	637 ± 176	0.111	−46 ± 144	−99 ± 135	0.535
CD_Glu_ (mmol/cm/s)	215 ± 26	237 ± 57	0.278	32 ± 66	8 ± 74	0.585
**Extracranial – anterior (ICA)**						
Velocity (cm/s)	33 ± 6	29 ± 6	**0.002**	−5 ± 4	−3 ± 4	0.546
Diameter (cm)	0.50 ± 0.04	0.55 ± 0.06	**0.001**	0.05 ± 0.02	0.05 ± 0.06	0.959
$\overset{\cdot }{Q}$ (mL/min)	196 ± 46	210 ± 76	0.183	10 ± 40	19 ± 28	0.667
CVCi (mL/min/mmHg)	2.19 ± 0.51	2.70 ± 0.91	**0.014**	0.38 ± 0.79	0.69 ± 0.08	0.341
CDO_2_ (mL/min)	39 ± 11	36 ± 15	0.263	−4 ± 9	−1 ± 4	0.573
CD_Glu_ (mmol/min/s)	1.2 ± 0.3	1.3 ± 0.5	0.131	0.1 ± 0.4	0.2 ± 0.2	0.806
E**xtracranial – posterior (VA)**						
Velocity (cm/s)	18 ± 3	18 ± 3	0.951	−1 ± 4	1 ± 1	0.418
Diameter (cm)	0.36 ± 0.05	0.38 ± 0.06	0.112	0.02 ± 0.04	0.01 ± 0.03	0.341
$\overset{\cdot }{Q}$ (mL/min)	59 ± 23	63 ± 22	0.331	5 ± 21	4 ± 6	0.947
CVCi (mL/min/mmHg)	0.66 ± 0.28	0.82 ± 0.29	**0.029**	0.14 ± 0.27	0.18 ± 0.14	0.779
CDO_2_ (mL/min)	12 ± 5	11 ± 4	0.529	−1 ± 5	0 ± 1	0.791
CD_Glu_ (mmol/min)	0.4 ± 0.2	0.4 ± 0.2	0.234	0.1 ± 0.2	0.0 ± 0.1	0.929
**Global**						
$\overset{\cdot }{Q}$ (mL/min)	509 ± 98	547 ± 156	0.178	31 ± 114	47 ± 52	0.746
CVCi (mL/min/mmHg)	5.71 ± 1.16	7.04 ± 1.90	**0.013**	1.05 ± 2.04	1.74 ± 0.36	0.414
CDO_2_ (mL/min)	101 ± 25	94 ± 32	0.315	−9 ± 28	−3 ± 7	0.619
CD_Glu_ (mmol/min)	3.1 ± 0.8	3.5 ± 1.0	0.136	0.4 ± 1.2	0.5 ± 0.5	0.868

MCAv: middle cerebral artery velocity; PCA: posterior cerebral artery velocity; ICA: internal carotid artery; VA: vertebral artery; CVCi: cerebrovascular conductance index; CDO_2_: cerebral delivery of oxygen; CD_Glu_: cerebral delivery of glucose.

Values are mean ± SD based on pooled data (normoxia vs. hypoxia, *n* = 12) and change (Δ hypoxia minus normoxia) in participants diagnosed with and without clinical acute mountain sickness (AMS+, *n* = 5 vs. AMS−, *n* = 7). Regional measures (MCA/PCA/ICA/VA) reflect unilateral calculations whereas global measurements reflect the sum of bilateral calculations.

Bold values indicate significance of *p*-values < 0.05.

#### Extracranial bioenergetics

Despite selective vasodilatation, hypoxia did not alter ${\overset{\cdot }{Q}}_{\mathrm{ICA}}$ due to a reduction in blood velocity (Table 3). Equally, hypoxia did not change ${\overset{\cdot }{Q}}_{\mathrm{VA}}$ whereas both ICA- and VA-CVCi were elevated (Table 3) due to the reduction in MAP (Supplementary Table 1). Hypoxia failed to alter both ICA- and VA-CDO_2_ and CD_Glu_ (Table 3). No differences were observed in AMS+ (Table 3).

#### Global bioenergetics

Hypoxia did not alter gCBF, gCDO_2_ or gCD_Glu_ (Table 3), whereas gCVCi increased (Table 3) due to a reduction in MAP (Supplementary Table 1). AMS+ did not alter hypoxia-induced changes in any of the metrics assessed (Table 3).

### Network physiology

#### VLFOs

**Figure 1.**
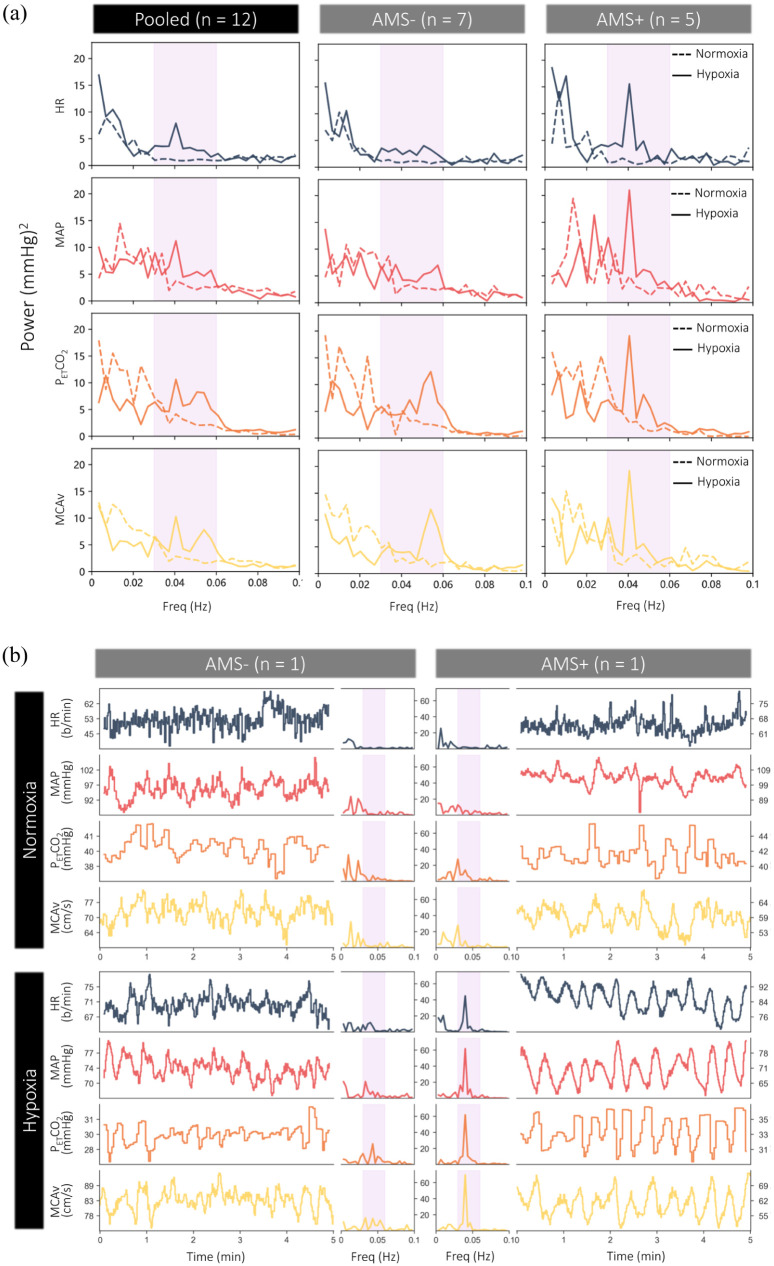
Very low frequency oscillations in hypoxia and acute mountain sickness: (a) pooled/subgroup responses to hypoxia and acute mountain sickness and (b) typical responses to hypoxia in a single participant with (AMS+) and without (AMS−) clinical AMS. Note that the single AMS+ participant selected was diagnosed with the most severe AMS (highest score). AMS: acute mountain sickness; HR: heart rate; MAP: mean arterial pressure; P_ET_CO_2_: end-tidal partial pressure of carbon dioxide; MCAv: middle cerebral artery velocity.

Compared to normoxia, hypoxia induced a marked elevation in VLFOs (0.03–0.06 Hz) that were readily observable in all physiological metrics and especially pronounced in AMS+ (Figure 1(a) and (b)). The strongest positive relationships were observed between AMS scores and RP of P_ET_O_2_ and P_ET_CO_2_–VLFOs in hypoxia (r = 0.725, *p* = 0.008 and r = 0.852, *p* < 0.001, respectively).

#### Physiological coupling

**Figure 2.**
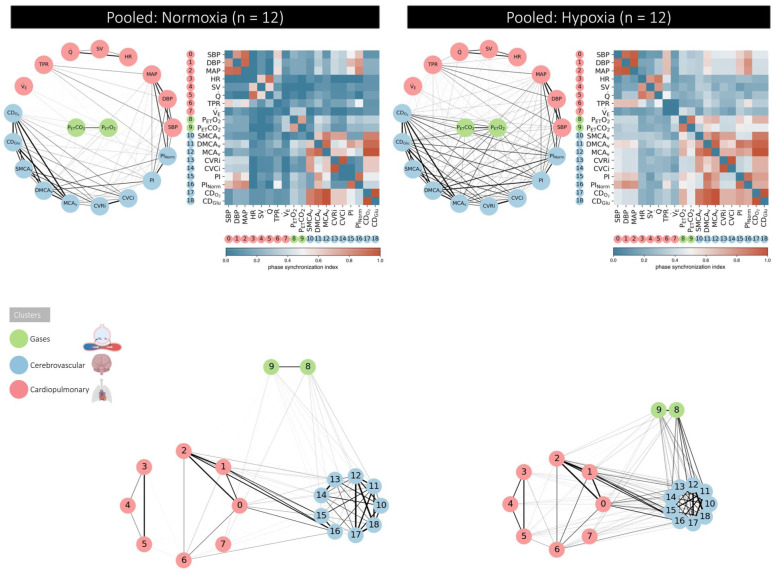
Physiological networks in hypoxia. Data averaged for all participants (*n* = 12) in normoxia and hypoxia. Illustrations include physiological networks, adjacency matrices and cluster coupling analyses. For the latter, distance within and between (physiological) networks is inversely proportional to interaction (connectivity) strength. The tighter, more aerodynamic the ‘virtual bicycle’, the stronger the connection(s).

**Figure 3.**
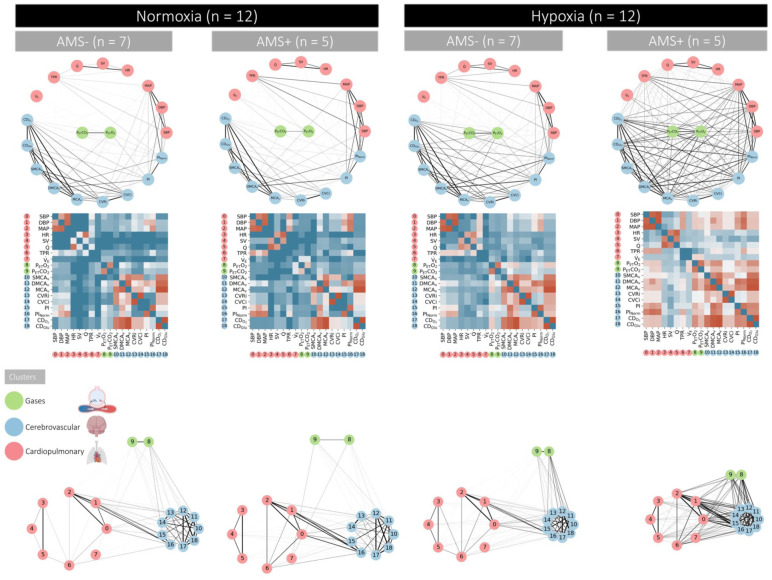
Physiological networks in acute mountain sickness. Participants diagnosed with (AMS+, *n* = 5) and without (AMS−, *n* = 7) clinical acute mountain sickness. Illustrations include physiological networks, adjacency matrices and cluster coupling analyses. For the latter, distance within and between (physiological) networks is inversely proportional to interaction (connectivity) strength. The tighter, more aerodynamic the ‘virtual bicycle’, the stronger the connection(s).

**Figure 4.**
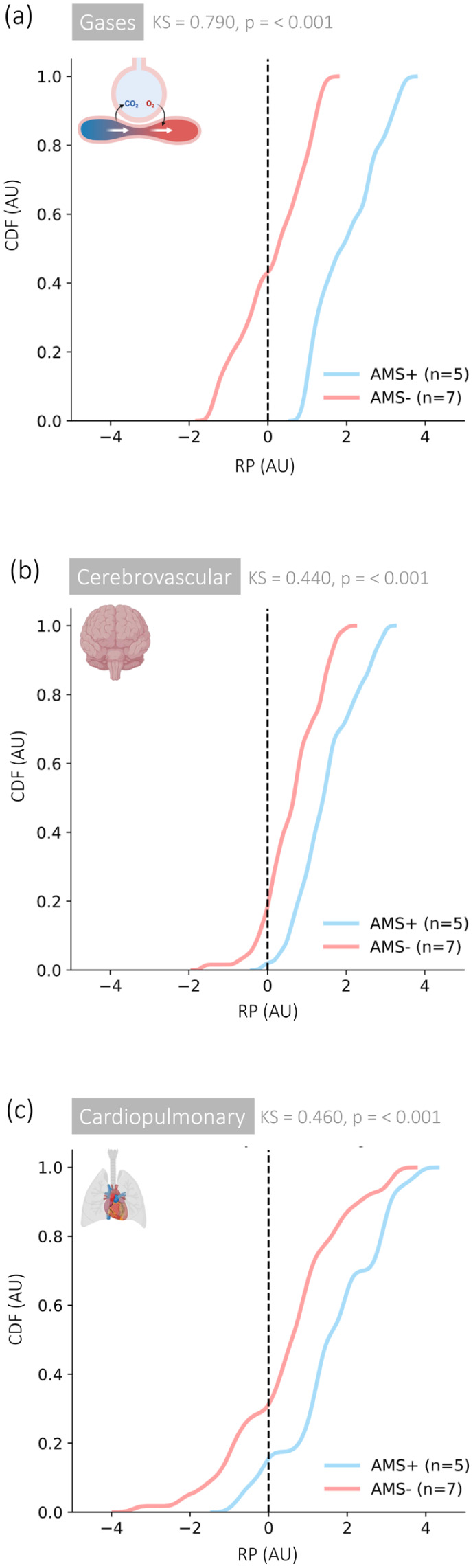
CDF of the RP of very low-frequency oscillations of signals associated with select physiological networks ((a) gases, (b) cerebrovascular and (c) cardiopulmonary) during the transition to hypoxia in participants with (AMS+, *n* = 5) and without (AMS−, *n* = 7) AMS. CDF plots obtained using kernel density estimation (Gaussian kernel with width *s* = 0.25). Note that a RP > 0 indicates that the amplitude of very low frequency oscillations increases during hypoxia. CDF: cumulative distribution function; RP: relative power; AMS: acute mountain sickness; AU, arbitrary units; KS: Kolmogorov–Smirnov test.

Hypoxia increased connectivity within and between physiological networks (Figure 2). This was most marked for the cerebrovascular cluster and especially pronounced in AMS+ (Figure 3). Cumulative distribution function plots highlighted that the respiratory gas cluster (P_ET_O_2_/P_ET_CO_2_) was the most dominant driving these connections (Figure 4).

### Additional analyses (potential carryover effects)

Retrospective analyses revealed no difference in hypoxia–normoxia Δ values between AB and BA sequences for molecular: A^·−^ (*p* = 0.313), plasma NO (*p* = 0.270), RBC NO (*p* = 0.719), total (plasma + RBC) NO (*p* = 0.422), S100B (*p* = 0.650), NSE (*p* = 0.902); haemodynamic: gCBF (*p* = 0.784) or clinical: LLS (*p* = 0.555), ESQ-C (*p* = 0.703) or VAS (*p* = 0.535) metrics.

## Discussion

In the field of integrative human physiology, it remains unclear how the cerebral, cardiac, pulmonary and metabolic systems collectively interact as a functional network to preserve cerebral bioenergetic homeostasis in hypoxia. Herein, we describe a conceptual framework to probe these dynamic interactions through the identification of physiological networks, highlighting three important findings. First and consistent with prior observations,^15^ cerebral substrate delivery was generally well maintained in hypoxia and AMS+ despite marked arterial hypoxaemia, conforming to the conservation of mass principle. Second, bioenergetic defence coincided with pronounced elevations in the spectral amplitude and synchronisation of oscillations within the VLF band (0.03–0.06 Hz) – these were prominent across all organ systems and especially marked within the cerebral network, likely reflecting hierarchical regulation. Third, and in stark contrast to our working hypothesis, VLFOs were further amplified in AMS+ and strongly linked to oscillations in P_ET_O_2_-P_ET_CO_2_ that were independent of exaggerated systemic OXNOS or structural destabilisation of the neurovascular unit. That cerebral bioenergetics were well maintained, and VLFOs further elevated and more functionally connected in AMS+, provocatively suggests that this cerebral syndrome, while characterised by debilitating symptomatology, may reflect a neuroprotective adaptive as opposed to pathologically maladaptive phenotype.

### VLFOs in hypoxia

Our analysis has unveiled the spontaneous and coordinated appearance of large amplitude systemic VLFOs during hypoxia, a finding that sheds new light on how physiological networks synchronise their activity under metabolic stress. Extending a prior NP study that employed transfer entropy to explore the functional connectivity between SpO_2_ and cardio-pulmonary time series in hypoxia,^47,48^ our findings reveal a previously unexplored aspect – hypoxia induces functional coupling across diverse physiological systems that better reflects the integrative adaptive phenotype underlying global cerebral bioenergetic defense.

Originally described by Lundberg as spontaneous fluctuations in intracranial pressure,^49^ VLFOs occur within the 0.02–0.07 Hz frequency band and have since been observed in a variety of physiological signals including cerebral blood velocity,^50^ arterial blood pressure,^51^ heart rate variability,^52^ electroencephalography,^53^ near-infrared spectroscopy,^54^ intraventricular cerebrospinal fluid flow^55^ and fMRI BOLD.^56^ While their precise origins remain unclear, VLFOs have been attributed to a complex interplay of neurogenic, metabolic and autonomic mechanisms.^57^ Indeed, it has long been recognised that temporal asynchrony between the sympathetic and parasympathetic components of the arterial baroreflex gives rise to a characteristic resonance frequency of ~0.1 Hz.^58^ Accordingly, it is plausible that resonance interactions among vascular beds may have contributed to the genesis of VLFOs. In addition, studies of dynamic cerebral autoregulation have shown that experimentally induced arterial pressure oscillations at comparable frequencies can causally drive corresponding oscillations in cerebral blood velocity, with behaviour closely resembling that of spontaneous VLFOs.^19,59,60^ This provides an important physiological basis to speculate that systemic haemodynamic drivers, particularly arterial pressure oscillations, may contribute to the emergence and propagation of the cerebral VLFOs observed herein, even if the present dataset was not designed to resolve mechanistic hierarchy within the broader network.

The physiological role of VLFOs remains equally elusive. While initially dismissed as ‘physiological noise’, the evolving interpretation suggests that these synchronised pulsations contribute to the coordinated defense of cerebrovascular homeostasis through optimisation of tissue oxygenation and interstitial fluid clearance.^61,62^ Previous NP studies have demonstrated that network coupling responds dynamically to physiological stress,^63^ including sleep states.^16,64,65^ Specifically, physiological networks exhibit low connectivity during deep (non-rapid eye movement) sleep, reflecting dominant parasympathetic tone and metabolic restoration associated with large, coupled oscillations in CSF flow observed at 0.05 Hz.^64^

In contrast, we observed more robust physiological coupling imposed by the systemic stress of acute hypoxia, which was particularly pronounced within the cerebrovascular and respiratory networks. This pattern is consistent with heightened sympathoexcitation – an established and highly conserved response to acute poikilocapnic hypoxemia, that is, initiated predominantly via peripheral chemoreceptor activation and propagated through integrated brainstem-autonomic circuits.^66^ As recently reviewed,^67^ acute hypoxia rapidly augments sympathetic nerve activity through convergent peripheral and central mechanisms that serve to preserve arterial pressure and systemic and cerebral O_2_ delivery. In this context, our findings suggest that network synchronisation is driven predominantly by respiratory gas dynamics (P_ET_O_2_ and P_ET_CO_2_), which may act to couple cerebrovascular and respiratory oscillations, constraining vasodilatory responses and stabilising cerebral perfusion. Equally, given the aforementioned pressure–flow coupling documented in dynamic autoregulation studies,^19,59,60^ it is plausible that oscillatory changes in arterial pressure interact with respiratory gas fluctuations to shape the cerebrovascular VLFO phenotype observed during hypoxia. The enhanced VLFO synchronisation observed across physiological systems may therefore reflect a coordinated homeostatic response that optimises cerebral substrate delivery and CO_2_ clearance under acute metabolic and hypoxic stress.

In support, clinical and computational modelling studies indicate that rhythmic oscillations of arterial pressure and flow (vasomotion) collectively enhance systemic O_2_ delivery by generating intermittent cyclical microvascular flow, increasing red blood cell velocity, with the most marked improvements observed at comparable frequencies to those observed herein (0.025–0.05 Hz).^62,68,69^ These VLFOs may also reduce precapillary O_2_ loss and further improve gas exchange through capillary distension, thereby decreasing diffusion distance and increasing surface area.^57^

Indeed, there may be a metabolic–endothelial contribution to the VLFOs observed in hypoxia, given that their amplification coincided with a systemic reduction in RBC NO. At face value, this observation is counterintuitive, as reduced vascular NO bioavailability has previously been implicated as a potential molecular risk factor for AMS subsequent to cerebral oxidative–nitrosative stress–mediated endothelial dysfunction.^15^ However, the physiological significance of this finding remains unresolved, as we did not isolate individual vasoactive moieties and are therefore unable to determine whether the reduction in RBC NO reflects diminished versus redistributed vascular NO bioactivity. In particular, we cannot distinguish between the principal mechanisms underpinning microvascular NO transport under hypoxic conditions, including (i) *S*-nitrosohaemoglobin formation with subsequent *S*-nitrosothiol release during the allosteric transition of Hb and (ii) ${NO}_{2}^{-}$ reduction by Hb to NO coupled with ATP release from deoxygenated RBCs.^28,70^

Moreover, while pharmacological augmentation of NO bioavailability (e.g. via dietary nitrate^71^ or *L*-arginine^72^ supplementation) might be expected to improve endothelial function and substrate delivery, such interventions have paradoxically been shown to exacerbate headache and increase AMS susceptibility, likely via trigeminovascular activation and/or NO-mediated increases in CBF and intracranial pressure.^71,73^ Within this context, the finding that VLFOs were further amplified in AMS despite reduced RBC NO underscores the complex and unresolved relationship between NO signalling, cerebrovascular regulation and hypoxic symptomatology, and cautions against simplistic therapeutic inference. Rather, these data support the need for targeted mechanistic and interventional studies to determine whether VLFO amplification represents a compensatory neuroprotective response operating independently of, or in concert with, NO-dependent pathways.

Caveats notwithstanding, that we failed to observe a systemic elevation in the ascorbate radical (A^·−^) during hypoxia consistent with previous observations.^74^ This tentatively argues against oxidative inactivation of NO by, for example, the superoxide anion (O_2^•^_^-^ ) and lipid centred radicals, to yield the reactant peroxynitrite (ONOO^−^; O_2_•^−^/L• + NO $\overset{10^{9}M/s}{\to }$ ONOO^−^).^75,76^ While limited evidence suggests that NO contributes, at least in part, to VLFOs (0.0095–0.021 Hz) previously documented in cutaneous laser doppler flow spectra,^77^ it is equally plausible that a reduction in NO bioavailability and corresponding loss of vasodilatory restraint on sympathetic outflow may promote exaggerated VLFOs – further highlighting the need for additional work to establish whether systemic OXNOS constitutes a major upstream driver of network coupling in hypoxia.

### VLFOs in AMS

Although AMS is traditionally considered a maladaptive response to hypoxia, our findings tentatively challenge this view. Consistent with prior observations,^14,15,42^ we failed to observe any molecular evidence of structural damage or destabilisation of the neurovascular unit (see “Experimental limitations and future directions” section). Second, the unexpected amplification of network synchronisation in AMS+ was generally associated with preserved cerebral bioenergetic function, except for a more marked reduction in anterior intracranial (MCA)-CDO_2_. This may prove an artefact related to the tendency towards a more marked hyperventilation-induced hypocapnia-mediated regional vasoconstriction, which cannot be confirmed using TCD ultrasound given its inability to measure changes in arterial diameter.

Regardless, the augmented VLFOs in AMS+ potentially reflect an adaptive neuroprotective response, rather than pathological rigidity. This interpretation is supported by recent work demonstrating that neuronal dynamics directly regulate CSF perfusion through the glymphatic system, the brain’s macroscopic waste clearance mechanism.^51,78^ Specifically, synchronised neuronal activity has been linked to the generation of large-amplitude ionic waves within the interstitial fluid that enhance CSF flow and clearance of metabolic byproducts, a process facilitated by the pulsatile forces exerted on elastic cerebral arteries that drive paravascular CSF movement.^79^ Inhibiting these neuronal oscillations disrupts brain clearance, whereas their artificial stimulation enhances metabolic waste removal.^51^ Set against the broader autoregulatory literature, it is conceivable that the amplified cerebral VLFOs reflect not merely epiphenomenal oscillatory behaviour, but a coordinated pressure–flow regulatory phenotype recruited under heightened hypoxic stress, potentially acting in concert with enhanced clearance-related pulsatility.

Given that hypoxia induces both systemic and cerebral OXNOS,^14,15,42^ it is plausible that the increased network synchronisation observed in AMS+ does indeed enhance brain clearance of noxious metabolites to confer hypoxic neuroprotection. Enhanced glymphatic or intramural periarterial drainage may also account for the observed reductions in S100B and NSE, attenuating regional spillover into the systemic circulation. However, excessive synchronisation can also serve to reduce network complexity and impair adaptive flexibility – mirroring patterns observed in epilepsy^80^ and migraine^81^ – highlighting the importance of distinguishing physiologically adaptive from pathologically maladaptive thresholds. Future studies should consider the functional integration of EEG monitoring, CSF flow imaging and arterio-jugular venous sampling to quantify transcerebral exchange kinetics and clarify whether hypoxia-induced VLFOs contribute directly to brain clearance mechanisms.

### Experimental limitations and future directions

Several limitations warrant consideration to provide a more balanced interpretation of our findings. The precise site(s) of origin of the observed VLFOs cannot be determined from the present data. Our NP approach relied on bivariate analyses and was therefore constrained to assessing phase synchronisation between pairs of oscillators; it cannot resolve coupling functions, interaction dynamics or identify the proximal generators of these oscillations. Although VLFOs were detectable across all measured cerebrovascular and cardiopulmonary signals – suggesting a systemic phenomenon with prominent expression within the cerebral network – this should not be interpreted as evidence of a uniquely brain-wide or brain-generated process. Similar low-frequency vasomotor oscillations have been reported in fMRI studies and linked to cerebrospinal fluid exchange and putative glymphatic mechanisms; however, our data are more consistent with VLFOs emerging from a complex interplay of neurogenic, metabolic and autonomic processes that propagate across multiple organ systems rather than arising from a single cerebral source. Future studies employing multivariate and directional approaches (e.g. transfer entropy or Granger causality), alongside spatially resolved neuroimaging, will be required to establish regional specificity and causal hierarchy among contributing oscillators.^82–84^

The absence of elevations in circulating S100B and NSE cannot definitively exclude subtle, spatially restricted or transient perturbations of the NVU or BBB. In prior work using arterio-jugular venous sampling in healthy humans, we have consistently observed a net transcerebral efflux of both S100B and NSE (jugular venous > arterial concentrations), indicating that the human brain continuously releases these proteins under physiological conditions.^15,33,85^ This supports the view that low-level peripheral detection reflects dynamic, homeostatic exchange across the BBB rather than structural NVU damage per se, consistent with the concept that the BBB is not a static barrier with occasional ‘leaks’ that may serve to clear excess protein or metabolites.^86^ However, interpretation is further constrained by delayed systemic kinetics (S100B ~60–120 min^87^; NSE up to ~30 h^88^) and ethical constraints that precluded serial blood sampling or arterio-jugular venous gradient measurements, which would be required to resolve the source, timing and trans-cerebral kinetics of protein release. Accordingly, our findings should be interpreted as indicating no detectable evidence of overt NVU injury rather than definitive absence of barrier disturbance. Indeed, rather than indicating injury, the observed reductions in circulating S100B and NSE are compatible with hypoxia-mediated tightening or stabilisation of the BBB.

Cerebral perfusion was quantified using established ultrasound-based indices which reflect ‘bulk’ conduit-artery flow and do not permit direct estimation of microvascular flow distribution, capillary recruitment or arteriovenous (a-v) capillary shunting. As such, any potential modulation of microvascular a-v shunt fraction by hypoxia – and its influence on inferred gCBF or substrate delivery – cannot be reliably quantified from the present dataset. Future studies integrating complementary approaches (e.g. arterial spin labelling and dynamic contrast-enhanced magnetic resonance imaging or positron emission tomography perfusion, combined with arterio-jugular venous sampling and/or microvascular oxygenation measures) will be required to resolve these effects.

While AMS-related symptoms (including cephalalgia) were recorded hourly to document their temporal evolution, the formal diagnosis of AMS was defined a priori and confirmed at the 6-h time point based on established threshold criteria. All multi-modal physiological measurements, including NP-derived VLFO metrics, were obtained exclusively at this 6-h time point and therefore coincide with confirmed clinical diagnosis rather than first symptom onset. As such, the present data do not permit determination of whether changes in VLFOs preceded, coincided with, or followed the initial emergence of AMS symptoms – an important temporal question that will require future studies with higher-resolution NP sampling across the exposure period.

Although prospective power calculations informed the study design, the relatively small sample size may have limited sensitivity to detect more subtle effects. The a priori focus on young male participants restricts generalisability, and future work should include female participants across the ageing continuum. Genetic background is recognised to contribute to inter-individual variability in hypoxic responses and may represent a relevant confounding factor.^89^ Although beyond the scope of the present study, future investigations incorporating genetic stratification – albeit challenging to implement – may provide important mechanistic insight. Our counterbalanced crossover design served to mitigate systematic order effects by distributing any residual carryover equally across (AB/BA) sequences and this was partly supported by the absence of differences in hypoxia–normoxia Δ values between AB and BA sequences for selected molecular, haemodynamic and clinical measures. However, given that a pre-exposure normoxic baseline was not obtained at the start of each period (due to cost constraints), residual post-exposure effects – particularly in participants completing hypoxia first (BA sequence) – cannot be unequivocally excluded.

Finally, our findings should be interpreted within the temporal context of hypoxic adaptation. Acclimation represents an important next step to determine whether the marked VLFO amplification observed here reflects an acute, non-acclimated defence response that attenuates or reorganises as systemic and cerebral oxygenation improve. Accordingly, longitudinal studies across the acclimation trajectory (e.g. sustained hypoxic exposure over several days) are likely to reveal progressive changes in VLFO amplitude and network connectivity; related datasets are currently under analysis, and chemoreflex-driven adjustments in cerebral blood flow and ventilation are likely to play an important role.^22^

## Conclusions

Alterations in VLF oscillatory dynamics and shifts in physiological network topology may help differentiate adaptive from maladaptive responses to hypoxia. Our findings emphasise the importance of evaluating not only individual system behaviour but also inter-system coupling, supporting the emerging paradigm that the integrity of physiological network architecture is central to maintaining cerebral bioenergetic homeostasis under hypoxic stress. More broadly, these observations suggest potential translational relevance for conditions characterised by acute or chronic arterial hypoxemia – such as myocardial infarction with cardiac arrest, ischaemic stroke, extracorporeal life support, chronic cardiopulmonary disease and ageing – where preservation, attenuation or potential loss or fragmentation of network oscillatory dynamics may reflect differing states of systemic autoregulatory competence.

## Supplemental Material

sj-docx-1-jcb-10.1177_0271678X261447119 – Supplemental material for Network oscillatory dynamics accompany cerebral bioenergetic defence in hypoxia Supplemental material, sj-docx-1-jcb-10.1177_0271678X261447119 for Network oscillatory dynamics accompany cerebral bioenergetic defence in hypoxia by Damian M Bailey, Benjamin S Stacey, Yaopeng Ma, Takuro Washio, Hayato Tsukamoto, Thomas S Owens, Thomas A Calverley, Lewis Fall, Christopher J Marley, Angelo Iannetelli, Takeshi Hashimoto, Soichi Ando, Shigehiko Ogoh, Nicola Marchi, Josip Butkovic, Ivan Mumlek, Brad Parry, Zvonomir Vrselja, James A Pawelczyk and Ronny P Bartsch in Journal of Cerebral Blood Flow & Metabolism

sj-pptx-2-jcb-10.1177_0271678X261447119 – Supplemental material for Network oscillatory dynamics accompany cerebral bioenergetic defence in hypoxia Supplemental material, sj-pptx-2-jcb-10.1177_0271678X261447119 for Network oscillatory dynamics accompany cerebral bioenergetic defence in hypoxia by Damian M Bailey, Benjamin S Stacey, Yaopeng Ma, Takuro Washio, Hayato Tsukamoto, Thomas S Owens, Thomas A Calverley, Lewis Fall, Christopher J Marley, Angelo Iannetelli, Takeshi Hashimoto, Soichi Ando, Shigehiko Ogoh, Nicola Marchi, Josip Butkovic, Ivan Mumlek, Brad Parry, Zvonomir Vrselja, James A Pawelczyk and Ronny P Bartsch in Journal of Cerebral Blood Flow & Metabolism

## References

[1] Bailey DM . Oxygen, evolution and redox signalling in the human brain; quantum in the quotidian. J Physiol 2019; 597: 15–28. 30315729 10.1113/JP276814 PMC6312417

[2] Bailey DM . Oxygen and brain death; back from the brink. Exp Physiol 2019; 104(12): 1769–1779. 31605408 10.1113/EP088005

[3] Volpi T Lee JJ Vlassenko AG , et al. The brain’s ‘dark energy’ puzzle upgraded: [^18^F]FDG uptake, delivery and phosphorylation, and their coupling with resting-state brain activity. J Cereb Blood Flow Metab 2025; 45(9): 1799–1815. 40370305 10.1177/0271678X251329707 PMC12081390

[4] Kety SS Schmidt CF . The effects of altered arterial tensions of carbon dioxide and oxygen on cerebral blood flow and cerebral oxygen consumption of normal young men. J Clin Invest 1948; 27(4): 484–492. 16695569 10.1172/JCI101995 PMC439519

[5] Willie CK Tzeng YC Fisher JA , et al. Integrative regulation of human brain blood flow. J Physiol 2014; 592(5): 841–859. 24396059 10.1113/jphysiol.2013.268953 PMC3948549

[6] Leacy JK Burns DP Jendzjowsky NG , et al. Characterizing the protective vasodilatory effects of hypobaric hypoxia on the neurovascular coupling response. J Cereb Blood Flow Metab 2025; 45(7): 1293–1309. 40079563 10.1177/0271678X251322348 PMC11907632

[7] Scheinberg P Stead EA . The cerebral blood flow in male subjects as measured by the nitrous oxide technique. Normal values for blood flow, oxygen utilization, glucose utilization, and peripheral resistance, with observations on the effect of tilting and anxiety. J Clin Invest 1949; 28(5 Pt 2): 1163–1171. 10.1172/JCI102150 PMC439673 16695788

[8] Caldwell HG Carr J Minhas JS , et al. Acid–base balance and cerebrovascular regulation. J Physiol 2021; 599(24): 5337–5359. 34705265 10.1113/JP281517

[9] Bailey DM Willie CK Hoiland RL , et al. Surviving without oxygen: how low can the human brain go?. High Alt Med Biol 2017; 18(1): 73–79. 28002687 10.1089/ham.2016.0081

[10] Bailey DM Bartsch P Knauth M , et al. Emerging concepts in acute mountain sickness and high-altitude cerebral edema: from the molecular to the morphological. Cell Mol Life Sci 2009; 66(22): 3583–3594. 19763397 10.1007/s00018-009-0145-9 PMC3085779

[11] Hackett PH Roach RC . High-altitude illness. N Engl J Med 2001; 345: 107–114. 11450659 10.1056/NEJM200107123450206

[12] Luks AM Swenson ER Bartsch P . Acute high-altitude sickness. Eur Respir Rev 2017; 26(143): 160096. 28143879 10.1183/16000617.0096-2016 PMC9488514

[13] Turner REF Gatterer H Falla M , et al. High-altitude cerebral edema: its own entity or end-stage acute mountain sickness?. J Appl Physiol 2021; 131(1): 313–325. 33856254 10.1152/japplphysiol.00861.2019

[14] Bailey DM Evans KA James PE , et al. Altered free radical metabolism in acute mountain sickness: implications for dynamic cerebral autoregulation and blood–brain barrier function. J Physiol 2009; 587(1): 73–85. 18936082 10.1113/jphysiol.2008.159855 PMC2670024

[15] Bailey DM Taudorf S Berg RMG , et al. Increased cerebral output of free radicals during hypoxia: implications for acute mountain sickness?. Am J Physiol Regul Integr Comp Physiol 2009; 297(5): R1283–R1292. 10.1152/ajpregu.00366.2009 19726713

[16] Bashan A Bartsch RP Kantelhardt JW , et al. Network physiology reveals relations between network topology and physiological function. Nat Commun 2012; 3: 702. 22426223 10.1038/ncomms1705 PMC3518900

[17] Ivanov PC . The new field of network physiology: building the human physiolome. Front Netw Physiol 2021; 1: 711778. 36925582 10.3389/fnetp.2021.711778 PMC10013018

[18] Williams JR . The Declaration of Helsinki and public health. Bull World Health Organ 2008; 86(8): 650–652. 18797627 10.2471/BLT.08.050955 PMC2649471

[19] Bailey DM Brugniaux JV Filipponi T , et al. Exaggerated systemic oxidative-inflammatory-nitrosative stress in chronic mountain sickness is associated with cognitive decline and depression. J Physiol 2019; 597(2): 611–629. 30397919 10.1113/JP276898 PMC6332753

[20] Marley CJ Davis D Brugniaux JV , et al. Post-prandial hyperlipidaemia impairs systemic vascular function and dynamic cerebral autoregulation in young and old male adults. J Nutr Physiol 2025; 2: 1–7.

[21] Wang J Brown MA Tam SH , et al. Effects of diet on measurement of nitric oxide metabolites. Clin Exp Pharmacol Physiol 1997; 24(6): 418–420. 9171946 10.1111/j.1440-1681.1997.tb01212.x

[22] Ogoh S Washio T Stacey BS , et al. Integrated respiratory chemoreflex-mediated regulation of cerebral blood flow in hypoxia: implications for oxygen delivery and acute mountain sickness. Exp Physiol 2021; 106(9): 1922–1938. 34318560 10.1113/EP089660

[23] Ando S Tsukamoto H Stacey BS , et al. Acute hypoxia impairs posterior cerebral bioenergetics and memory in man. Exp Physiol 2023; 108(12): 1516–1530. 37898979 10.1113/EP091245 PMC10988469

[24] Davison GW Ashton T McEneny J , et al. Critical difference applied to exercise-induced oxidative stress: the dilemma of distinguishing biological from statistical change. J Physiol Biochem 2012; 68(3): 377–384. 22298153 10.1007/s13105-012-0149-z

[25] Rose GA Davies RG Davison GW , et al. The cardiopulmonary exercise test grey zone; optimising fitness stratification by application of critical difference. Br J Anaesth 2018; 120(6): 1187–1194. 29793585 10.1016/j.bja.2018.02.062

[26] Bailey DM Laneelle D Trihan JE , et al. Gravitational transitions increase posterior cerebral perfusion and systemic oxidative-nitrosative stress: implications for neurovascular unit integrity. Neuroscience 2020; 441: 142–160. 32502571 10.1016/j.neuroscience.2020.05.048

[27] Bailey DM Rasmussen P Evans KA , et al. Hypoxia compounds exercise-induced free radical formation in humans; partitioning contributions from the cerebral and femoral circulation. Free Radic Biol Med 2018; 124: 104–113. 29859345 10.1016/j.freeradbiomed.2018.05.090

[28] Bailey DM Rasmussen P Overgaard M , et al. Nitrite and *S*-nitrosohemoglobin exchange across the human cerebral and femoral circulation: relationship to basal and exercise blood flow responses to hypoxia. Circulation 2017; 135(2): 166–176. 27881556 10.1161/CIRCULATIONAHA.116.024226

[29] Rogers SC Khalatbari A Gapper PW , et al. Detection of human red blood cell-bound nitric oxide. J Biol Chem 2005; 280(29): 26720–26728. 15879596 10.1074/jbc.M501179200

[30] Pinder AG Rogers SC Khalatbari A , et al. The measurement of nitric oxide and its metabolites in biological samples by ozone-based chemiluminescence. Methods Mol Biol 2009; 476: 10–27. 10.1007/978-1-59745-129-1_2 19157006

[31] Janigro D Bailey DM Lehmann S , et al. Peripheral blood and salivary biomarkers of blood–brain barrier permeability and neuronal damage: clinical and applied concepts. Front Neurol 2020; 11: 577312. 33613412 10.3389/fneur.2020.577312 PMC7890078

[32] Michetti F D’Ambrosi N Toesca A , et al. The S100B story: from biomarker to active factor in neural injury. J Neurochem 2019; 148(2): 168–187. 30144068 10.1111/jnc.14574

[33] Bailey DM Bain AR Hoiland RL , et al. Hypoxemia increases blood–brain barrier permeability during extreme apnea in humans. J Cereb Blood Flow Metab 2022; 42(6): 1120–1135. 35061562 10.1177/0271678X221075967 PMC9121528

[34] Pahlman S Esscher T Bergvall P , et al. Purification and characterization of human neuron-specific enolase: radioimmunoassay development. Tumour Biol 1984; 5(2): 127–139. 6505548

[35] Wesseling KH Jansen JR Settels JJ , et al. Computation of aortic flow from pressure in humans using a nonlinear, three-element model. J Appl Physiol 1993; 74(5): 2566–2573. 8335593 10.1152/jappl.1993.74.5.2566

[36] Woodman RJ Playford DA Watts GF , et al. Improved analysis of brachial artery ultrasound using a novel edge-detection software system. J Appl Physiol 2001; 91(2): 929–937. 11457812 10.1152/jappl.2001.91.2.929

[37] Willie CK Macleod DB Shaw AD , et al. Regional brain blood flow in man during acute changes in arterial blood gases. J Physiol 2012; 590(14): 3261–3275. 22495584 10.1113/jphysiol.2012.228551 PMC3459041

[38] Friend AT Rogan M Rossetti GMK , et al. Bilateral regional extracranial blood flow regulation to hypoxia and unilateral duplex ultrasound measurement error. Exp Physiol 2021; 106(7): 1535–1548. 33866627 10.1113/EP089196

[39] Roach RC Hackett PH Oelz O , et al. The 2018 Lake Louise Acute Mountain Sickness score. High Alt Med Biol 2018; 19(1): 4–6. 29583031 10.1089/ham.2017.0164 PMC6191821

[40] Sampson JB Cymerman A Burse RL , et al. Procedures for the measurement of acute mountain sickness. Aviat Space Environ Med 1983; 54(12 Pt 1): 1063–1073. 6661120

[41] Iversen HK Olesen J Tfelt-Hansen P . Intravenous nitroglycerin as an experimental model of vascular headache. Basic characteristics. Pain 1989; 38(1): 17–24. 2506503 10.1016/0304-3959(89)90067-5

[42] Bailey DM Roukens R Knauth M , et al. Free radical-mediated damage to barrier function is not associated with altered brain morphology in high-altitude headache. J Cereb Blood Flow Metab 2006; 26(1): 99–111. 15959459 10.1038/sj.jcbfm.9600169

[43] Bartsch RP Liu KK Bashan A , et al. Network physiology: how organ systems dynamically interact. PLoS One 2015; 10(11): e0142143. 10.1371/journal.pone.0142143 PMC4640580 26555073

[44] Rosenblum M Pikovsky A Kurths J , et al. Phase synchronization: from theory to data analysis. In: Moss F Gielen S (eds.) Neuro-informatics and neural modelling, vol. 4. Amsterdam: Elsevier, 2001, pp.279–321.

[45] Xu L Chen Z Hu K , et al. Spurious detection of phase synchronization in coupled nonlinear oscillators. Phys Rev E 2006; 73(6 Pt 2): 065201. 10.1103/PhysRevE.73.065201 16906897

[46] Virtanen P Gommers R Oliphant TE , et al. SciPy 1.0: fundamental algorithms for scientific computing in Python. Nat Methods 2020; 17(3): 261–272. 32015543 10.1038/s41592-019-0686-2 PMC7056644

[47] Jiang Y Costello JT Williams TB , et al. A network physiology approach to oxygen saturation variability during normobaric hypoxia. Exp Physiol 2021; 106(1): 151–159. 32643311 10.1113/EP088755

[48] Morandotti C Rigny L Williams TB , et al. Non-invasive assessment of integrated cardiorespiratory network dynamics after physiological stress in humans. J Physiol 2025. 10.1113/JP288939 40623438

[49] Lundberg N . Continuous recording and control of ventricular fluid pressure in neurosurgical practice. Acta Psychiatr Scand Suppl 1960; 36(149): 1–193. 13764297

[50] Muller T Reinhard M Oehm E , et al. Detection of very low-frequency oscillations of cerebral haemodynamics is influenced by data detrending. Med Biol Eng Comput 2003; 41(1): 69–74. 12572750 10.1007/BF02343541

[51] Julien C . An update on the enigma of Mayer waves. Cardiovasc Res 2020; 116(14): e210–e211. 10.1093/cvr/cvz327 31865368

[52] Bigger JT Jr Fleiss JL Steinman RC , et al. Frequency domain measures of heart period variability and mortality after myocardial infarction. Circulation 1992; 85(1): 164–171. 1728446 10.1161/01.cir.85.1.164

[53] Helps S James C Debener S , et al. Very low frequency EEG oscillations and the resting brain in young adults: a preliminary study of localisation, stability and association with symptoms of inattention. J Neural Transm 2008; 115(2): 279–285. 17994187 10.1007/s00702-007-0825-2

[54] Gruszecka A Waskow M Malkiewicz MA , et al. Mild poikilocapnic hypoxia increases very low frequency haemoglobin oxygenation oscillations in prefrontal cortex. Biol Res 2021; 54(1): 39. 34906247 10.1186/s40659-021-00362-2 PMC8669425

[55] Strik C Klose U Kiefer C , et al. Slow rhythmic oscillations in intracranial CSF and blood flow: registered by MRI. Acta Neurochir Suppl 2002; 81: 139–142. 12168286 10.1007/978-3-7091-6738-0_36

[56] Biswal B Yetkin FZ Haughton VM , et al. Functional connectivity in the motor cortex of resting human brain using echo-planar MRI. Magn Reson Med 1995; 34(4): 537–541. 8524021 10.1002/mrm.1910340409

[57] Anderson GK Rickards CA . The potential therapeutic benefits of low frequency haemodynamic oscillations. J Physiol 2022; 600(17): 3905–3919. 35883272 10.1113/JP282605 PMC9444954

[58] deBoer RW Karemaker JM Strackee J . Hemodynamic fluctuations and baroreflex sensitivity in humans: a beat-to-beat model. Am J Physiol 1987; 253(3 Pt 2): H680–H689. 10.1152/ajpheart.1987.253.3.H680 3631301

[59] Claassen JA Levine BD Zhang R . Dynamic cerebral autoregulation during repeated squat-stand maneuvers. J Appl Physiol 2009; 106(1): 153–160. 18974368 10.1152/japplphysiol.90822.2008 PMC2636935

[60] Claassen J Thijssen DHJ Panerai RB , et al. Regulation of cerebral blood flow in humans: physiology and clinical implications of autoregulation. Physiol Rev 2021; 101(4): 1487–1559. 33769101 10.1152/physrev.00022.2020 PMC8576366

[61] Jiang-Xie LF Drieu A Bhasiin K , et al. Neuronal dynamics direct cerebrospinal fluid perfusion and brain clearance. Nature 2024; 627(8002): 157–164. 38418877 10.1038/s41586-024-07108-6 PMC12054998

[62] Rucker M Strobel O Vollmar B , et al. Vasomotion in critically perfused muscle protects adjacent tissues from capillary perfusion failure. Am J Physiol Heart Circ Physiol 2000; 279(2): H550–H558. 10.1152/ajpheart.2000.279.2.H550 10924053

[63] Stamatis A Morgan GB Reyes JC . Dynamic interactions of physiological systems during competitive gaming: insights from network physiology – case report. Front Netw Physiol 2024; 4: 1438073. 39324076 10.3389/fnetp.2024.1438073 PMC11422231

[64] Fultz NE Bonmassar G Setsompop K , et al. Coupled electrophysiological, hemodynamic, and cerebrospinal fluid oscillations in human sleep. Science 2019; 366(6465): 628–631. 31672896 10.1126/science.aax5440 PMC7309589

[65] Xie L Kang H Xu Q , et al. Sleep drives metabolite clearance from the adult brain. Science 2013; 342(6156): 373–377. 24136970 10.1126/science.1241224 PMC3880190

[66] Tymko MM Young D Vergel D , et al. The effect of hypoxemia on muscle sympathetic nerve activity and cardiovascular function: a systematic review and meta-analysis. Am J Physiol Regul Integr Comp Physiol 2023; 325(5): R474–R489. 10.1152/ajpregu.00021.2023 37642283

[67] Simpson LL Stembridge M Siebenmann C , et al. Mechanisms underpinning sympathoexcitation in hypoxia. J Physiol 2024; 602(21): 5485–5503. 38533641 10.1113/JP284579

[68] Tsai AG Intaglietta M . Evidence of flowmotion induced changes in local tissue oxygenation. Int J Microcirc Clin Exp 1993; 12(1): 75–88. 8473071

[69] Goldman D Popel AS . A computational study of the effect of vasomotion on oxygen transport from capillary networks. J Theor Biol 2001; 209(2): 189–199. 11401461 10.1006/jtbi.2000.2254

[70] Hoiland RL MacLeod DB Stacey BS , et al. Hemoglobin and cerebral hypoxic vasodilation in humans: evidence for nitric oxide-dependent and S-nitrosothiol mediated signal transduction. J Cereb Blood Flow Metab 2023; 43(9): 1519–1531. 37042194 10.1177/0271678X231169579 PMC10414015

[71] Rossetti GMK Macdonald JH Wylie LJ , et al. Dietary nitrate supplementation increases acute mountain sickness severity and sense of effort during hypoxic exercise. J Appl Physiol 2017; 123(4): 983–992. 28684588 10.1152/japplphysiol.00293.2017

[72] Mansoor JK Morrissey BM Walby WF , et al. L-arginine supplementation enhances exhaled NO, breath condensate VEGF, and headache at 4,342 m. High Alt Med Biol 2005; 6(4): 289–300. 16351563 10.1089/ham.2005.6.289

[73] Bailey DM Taudorf S Berg RM , et al. Transcerebral exchange kinetics of nitrite and calcitonin gene-related peptide in acute mountain sickness: evidence against trigeminovascular activation?. Stroke 2009; 40(6): 2205–2208. 19359638 10.1161/STROKEAHA.108.543959

[74] Stacey BS Marley CJ Tsukamoto H , et al. Phosphodiesterase inhibition restores hypoxia-induced cerebrovascular dysfunction subsequent to improved systemic redox homeostasis: a randomized, double-blind, placebo-controlled crossover study. J Cereb Blood Flow Metab 2025; 45(7): 1343–1356. 39862172 10.1177/0271678X251313747 PMC11765346

[75] Bailey DM Young IS McEneny J , et al. Regulation of free radical outflow from an isolated muscle bed in exercising humans. Am J Physiol Heart Circ Physiol 2004; 287: H1689–H1699. 10.1152/ajpheart.00148.2004 15155256

[76] Nauser T Koppenol WH . The rate constant of the reaction of superoxide with nitrogen monoxide: approaching the diffusion limit. J Phys Chem A 2002; 106: 4084–4086.

[77] Stewart JM Taneja I Goligorsky MS , et al. Noninvasive measure of microvascular nitric oxide function in humans using very low-frequency cutaneous laser Doppler flow spectra. Microcirculation 2007; 14(3): 169–180. 17454670 10.1080/10739680601139179 PMC4513357

[78] Vakhtin AA Lin HC Pirio Richardson SE , et al. Harnessing brain rhythms to activate the glymphatic pathway via controlled breathing and intermittent CO_2_. J Cereb Blood Flow Metab 2025; 46: 444–455. 41355053 10.1177/0271678X251399120 PMC12685709

[79] Wen Q Muskat J Babbs CF , et al. Dynamic diffusion-weighted imaging of intracranial cardiac impulse propagation along arteries to arterioles in the aging brain. J Cereb Blood Flow Metab 2025; 45(8): 1519–1530. 39947901 10.1177/0271678X251320902 PMC11826823

[80] Moridani MK Farhadi H . Heart rate variability as a biomarker for epilepsy seizure prediction. Bratisl Lek Listy 2017; 118(1): 3–8. 28127975 10.4149/BLL_2017_001

[81] Hodkinson DJ Wilcox SL Veggeberg R , et al. Increased amplitude of thalamocortical low-frequency oscillations in patients with migraine. J Neurosci 2016; 36(30): 8026–8036. 27466345 10.1523/JNEUROSCI.1038-16.2016 PMC4961783

[82] Scagliarini T Sparacino L Faes L , et al. Gradients of O-information highlight synergy and redundancy in physiological applications. Front Netw Physiol 2023; 3: 1335808. 38264338 10.3389/fnetp.2023.1335808 PMC10803408

[83] Pichot V Corbier C Chouchou F . The contribution of granger causality analysis to our understanding of cardiovascular homeostasis: from cardiovascular and respiratory interactions to central autonomic network control. Front Netw Physiol 2024; 4: 1315316. 39175608 10.3389/fnetp.2024.1315316 PMC11338816

[84] West BJ . Complexity synchronization in living matter: a mini review. Front Netw Physiol 2024; 4: 1379892. 38831910 10.3389/fnetp.2024.1379892 PMC11145412

[85] Bain AR Ainslie PN Hoiland RL , et al. Competitive apnea and its effect on the human brain: focus on the redox regulation of blood–brain barrier permeability and neuronal–parenchymal integrity. FASEB J 2018; 32(4): 2305–2314. 29191963 10.1096/fj.201701031R

[86] Bargerstock E Puvenna V Iffland P , et al. Is peripheral immunity regulated by blood–brain barrier permeability changes?. PLoS One 2014; 9(7): e101477. 10.1371/journal.pone.0101477 PMC4079719 24988410

[87] Thelin EP Zeiler FA Ercole A , et al. Serial sampling of serum protein biomarkers for monitoring human traumatic brain injury dynamics: a systematic review. Front Neurol 2017; 8: 300. 28717351 10.3389/fneur.2017.00300 PMC5494601

[88] Rundgren M Cronberg T Friberg H , et al. Serum neuron specific enolase – impact of storage and measuring method. BMC Res Notes 2014; 7: 726. 25319200 10.1186/1756-0500-7-726 PMC4216829

[89] Lancaster G Debevec T Millet GP , et al. Relationship between cardiorespiratory phase coherence during hypoxia and genetic polymorphism in humans. J Physiol 2020; 598(10): 2001–2019. 31957891 10.1113/JP278829 PMC7317918

