# Supplementary material for: Network oscillatory dynamics accompany cerebral bioenergetic defence in hypoxia
Source: J Cereb Blood Flow Metab. 2026 Jun 4:0271678X261447119. Online ahead of print. doi: 10.1177/0271678X261447119 (PMC13236721; doi:10.1177/0271678X261447119)
Supplement: sj-docx-1-jcb-10.1177_0271678X261447119 – Supplemental material for Network oscillatory dynamics accompany cerebral bioenergetic defence in hypoxia [file sj-docx-1-jcb-10.1177_0271678X261447119.docx]

**Supplementary material**

**Legend**

**Supplementary Figure 1.** Temporal evolution of acute mountain sickness and headache symptom scores

Values are mean (horizontal bars connected by stippled lines) ± SD based on pooled data (A-C: normoxia vs. hypoxia, n = 12) in participants diagnosed with (AMS+, n = 5) and without (AMS-, n = 7) clinical acute mountain sickness (D-F). Grey stippled lines highlight cut-off scores for clinical diagnosis of AMS for LL and ESQ-C scoring systems. LLS, Lake Louise score; ESQ-C, Environmental Symptoms Questionnaire-Cerebral; VAS, visual analogue scale; AU, arbitrary units. †different (P < 0.05) as a function of condition (nomoxia vs. hypoxia) or state (AMS+ vs. AMS-). Created in BioRender.
