# Supplementary material for: Network oscillatory dynamics accompany cerebral bioenergetic defence in hypoxia
Source: J Cereb Blood Flow Metab. 2026 Jun 4:0271678X261447119. Online ahead of print. doi: 10.1177/0271678X261447119 (PMC13236721; doi:10.1177/0271678X261447119)
Supplement: sj-pptx-2-jcb-10.1177_0271678X261447119 – Supplemental material for Network oscillatory dynamics accompany cerebral bioenergetic defence in hypoxia [file sj-pptx-2-jcb-10.1177_0271678X261447119.pptx]

## Slide 1
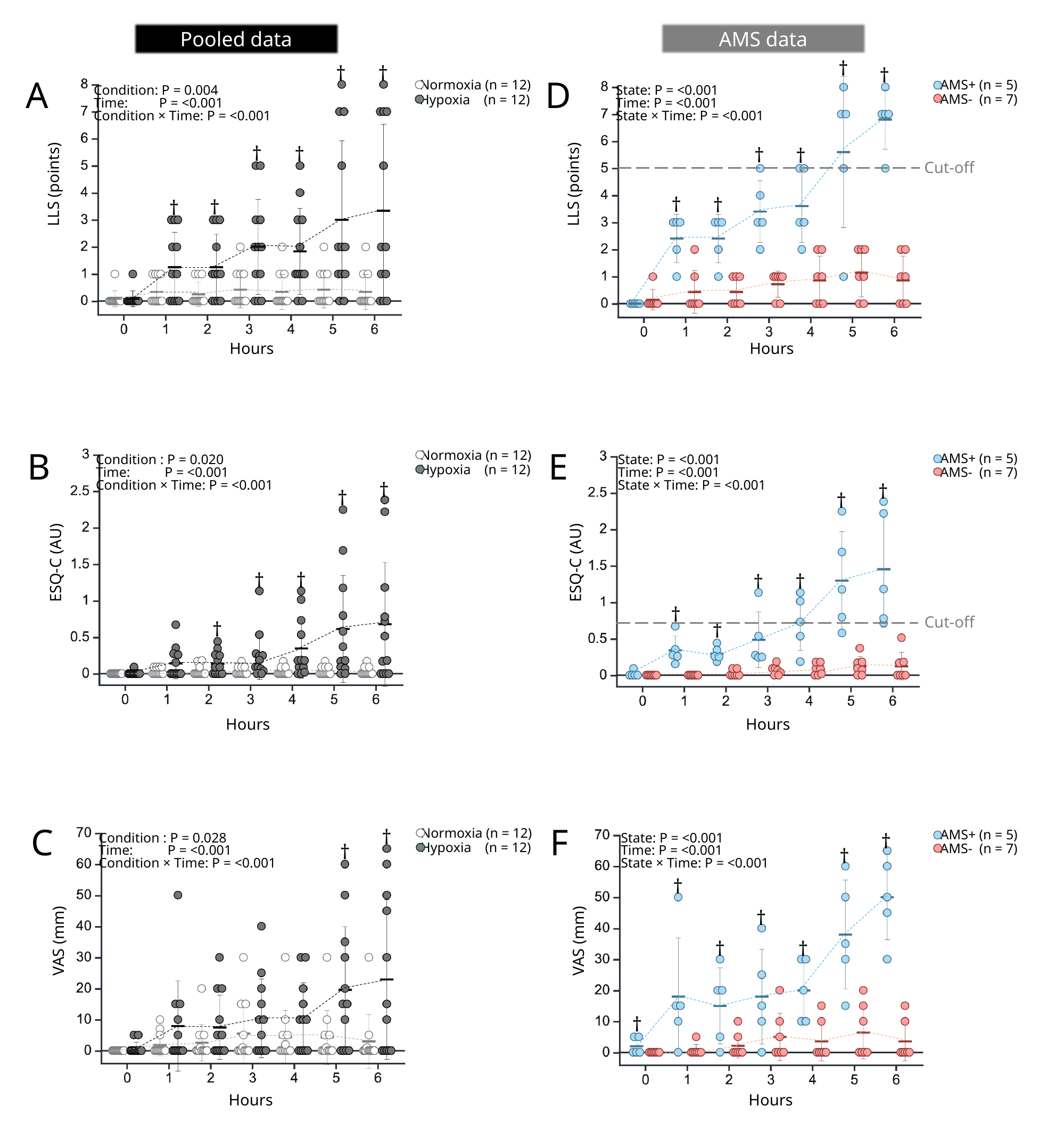

AMS data
Pooled data
†
†
†
†
A
D
Normoxia (n = 12)
AMS+ (n = 5)
Condition: P = 0.004
Time: P = <0.001
Condition × Time: P = <0.001
State: P = <0.001
Time: P = <0.001
State × Time: P = <0.001
AMS- (n = 7)
Hypoxia (n = 12)
†
†
†
†
Cut-off
LLS (points)
LLS (points)
†
†
†
†
Hours
Hours
B
E
Normoxia (n = 12)
AMS+ (n = 5)
Condition : P = 0.020
Time: P = <0.001
Condition × Time: P = <0.001
State: P = <0.001
Time: P = <0.001
State × Time: P = <0.001
Hypoxia (n = 12)
AMS- (n = 7)
†
†
†
†
ESQ-C (AU)
ESQ-C (AU)
†
†
†
†
†
Cut-off
†
†
Hours
Hours
C
F
†
Normoxia (n = 12)
AMS+ (n = 5)
†
Condition : P = 0.028
Time: P = <0.001
Condition × Time: P = <0.001
State: P = <0.001
Time: P = <0.001
State × Time: P = <0.001
†
Hypoxia (n = 12)
AMS- (n = 7)
†
†
†
VAS (mm)
VAS (mm)
†
†
†
Hours
Hours
